# Tuberculosis in older adults (≥ 65 years): Global trends, sex differences, and regional variations, 2000–2023

**DOI:** 10.1186/s12877-025-06934-1

**Published:** 2026-01-10

**Authors:** Josef Yayan, Kurt Rasche

**Affiliations:** 1 Division of Pulmonary, Allergy, and Sleep Medicine, Department of Internal Medicine, HELIOS Clinic Wuppertal, Witten/Herdecke University, Witten, Germany; 2 Division of Pulmonary, Allergy, and Sleep Medicine, Department of Internal Medicine, HELIOS Clinic Wuppertal Witten/Herdecke University, Heusnerstr. 40, Wuppertal, 42283 Germany

**Keywords:** Tuberculosis, Older adults (≥ 65 years), Incidence, Mortality, Trends 2000–2023, Global burden, Sex differences

## Abstract

**Background:**

Older adults (≥ 65 years) are at increased risk for tuberculosis (TB), but global burden estimates in this population remain limited. This study analyzes long-term trends from 2000 to 2023 and assesses TB incidence, mortality, and sex-specific differences among older adults.

**Methods:**

Age- and sex-specific TB case data were obtained from the World Health Organization (WHO) Global TB Database and United Nations population estimates. TB incidence and mortality per 100,000 population were calculated for individuals aged ≥ 65 years. Rate ratios (RR) comparing adults ≥ 65 years with the WHO standard adult reference group (individuals aged 15–64 years) were computed. Geographic and sex-specific differences were analyzed using descriptive statistics and choropleth maps.

**Results:**

In 2023, individuals aged ≥ 65 years accounted for 21% of global TB cases and 23% of TB deaths. Between 2000 and 2023, TB cases in this age group increased from 610,000 to 947,000, despite declining global TB incidence overall. Rate ratios for incidence and mortality were elevated across all WHO regions, with the highest rate ratios in the Western Pacific and South-East Asia. Men had approximately twice the incidence of TB compared with women. Geographic distribution showed highest burden in South-East Asia, sub-Saharan Africa (influenced by the human immunodeficiency virus–TB co-epidemic), and Eastern Europe (driven partly by multidrug-resistant -TB).

**Conclusions:**

TB burden among older adults is substantial and has increased over the past two decades. Clear sex-specific disparities persist. Age- and sex-tailored public health strategies—including targeted screening, integration of comorbidity management, and strengthened surveillance—are essential to reduce TB-related morbidity and mortality in this vulnerable population.

## Introduction

Tuberculosis (TB) remains a leading cause of infectious morbidity and mortality worldwide, despite significant progress in global TB control over the past decades [1, 2]. Older adults (defined according to World Health Organization (WHO) and United Nations (UN) demographic standards as individuals aged ≥ 65 years) represent a particularly vulnerable population due to age-related immunosenescence, a higher prevalence of comorbidities, and increased susceptibility to severe disease [3–5]. In many high- and middle-income countries, the proportion of TB cases occurring in older adults is steadily rising, reflecting both demographic shifts and successful TB control in younger populations [6, 7].

Age-specific analyses of TB burden are essential for understanding epidemiological trends, guiding targeted interventions, and prioritizing resource allocation [8, 9]. However, most global TB estimates focus on the overall population or younger age groups, and the epidemiology among older adults remains underexplored [10]. Furthermore, sex-specific differences in TB incidence and mortality are often overlooked, even though men generally exhibit higher TB risk compared with women [11–13]. In addition, regional factors such as the human immunodeficiency virus (HIV)–TB syndemic in sub-Saharan Africa, the high prevalence of diabetes in South-East Asia, and the substantial burden of multidrug-resistant TB (MDR-TB) in Eastern Europe contribute to marked geographic heterogeneity in TB outcomes among older adults.

A comprehensive assessment of TB incidence and mortality in older adults, stratified by sex, country, and region, is critical to inform age-appropriate public health strategies. This study therefore examines global and regional trends in TB burden from 2000 to 2023, estimates rate ratios comparing older adults with the WHO standard adult reference population (aged 15–64 years), and evaluates sex-specific differences using the most recent WHO TB data and United Nations population estimates [14–16].

## Materials and methods

### Data sources

We obtained age- and sex-specific tuberculosis (TB) case data from the World Health Organization (WHO) Global Tuberculosis Database. Data included reported TB incidence and mortality counts for all countries between 2000 and 2023. Population estimates for individuals aged ≥ 65 years were retrieved from the United Nations World Population Prospects 2016 revision (latest available estimates for the population aged ≥ 65 years by country). The definition of older adults (≥ 65 years) follows WHO and UN demographic standards used in global ageing and epidemiologic surveillance. Where sex-specific population aged ≥ 65 years data were unavailable, total population counts were used as a proxy for calculating incidence rates by sex.

### Study population

The study focused on individuals aged ≥ 65 years. This age group was selected because it represents the internationally recognized threshold for older adulthood in global demographic and epidemiologic monitoring. For comparative analyses, the younger adult population (15–64 years) was used as the reference group. This reference category corresponds to the WHO standard adult population used in TB surveillance and reflects the primary age group contributing to TB transmission. TB cases were analyzed globally and stratified by WHO region and country. Sex-specific analyses were conducted for males and females aged ≥ 65 years.

### Outcome measures

The primary outcomes were:

1. TB incidence per 100,000 population among individuals aged ≥ 65 years.
2. TB mortality per 100,000 population among individuals aged ≥ 65 years.

Secondary outcomes included:

- Rate ratios comparing incidence and mortality in ≥ 65-year-olds versus 15–64-year-olds.
- Sex-specific incidence among older adults.
- Temporal trends in TB burden from 2000 to 2023.

### Data processing and analysis

Age-specific TB cases were summed across sexes to calculate total cases in each age group. For sex-specific analyses, male and female cases were kept separate. Incidence rates were calculated as:

Incidence per 100,000 = (Number of TB cases/Population aged ≥ 65) × 100,000.

In cases where population data for 2023 were unavailable, the most recent available estimates (2016) were used as a proxy. To compare tuberculosis burden between age groups, we calculated incidence and mortality rate ratios (RR) by dividing age-specific rates in adults aged ≥ 65 years by those in individuals aged 15–64 years. In this population-based ecological analysis, the RR represents an age-specific rate ratio rather than an individual-level relative risk, as no individual-level follow-up or exposure data were available. This approach is widely used in WHO tuberculosis surveillance and Global Burden of Disease age-specific analyses [1]. 95% confidence intervals for RR were calculated using log-transformed Poisson standard errors.

### Statistical analysis

Descriptive statistics were calculated for TB incidence and mortality across years, regions, and countries. 95% confidence intervals (CIs) were used to assess the precision of estimates for sex-specific analyses. Geographic variation in TB incidence among older adults was visualized using choropleth maps, with a logarithmic scale applied to better display differences across countries. An additional global map was created to display rate ratios (≥ 65 vs. 15–64 years). 95% CIs for incidence rates were calculated using the Poisson approximation. All statistical analyses and data visualizations were performed using Python (version 3.10) with the pandas, numpy, and matplotlib libraries, ensuring reproducibility of results. All reported rate ratios should be interpreted as population-level comparisons of age-specific tuberculosis rates rather than estimates of individual risk.

We additionally evaluated long-term trends from 2000 to 2023 to reflect demographic changes, shifts in TB epidemiology, and potential disruptions during the COVID-19 pandemic period.

## Results

Between 2000 and 2023, the global population aged ≥ 65 years increased substantially, accompanied by marked changes in tuberculosis (TB) burden. During this period, the number of older adults worldwide rose from 419.8 million to 771.2 million, representing an 84% increase. The absolute number of TB cases and deaths among older adults rose steadily, from 610,000 cases and 162,000 deaths in 2000 to 947,000 cases and 227,000 deaths in 2023, despite overall global declines in TB incidence and mortality in younger age groups. In 2023, individuals aged ≥ 65 years accounted for 21% of global TB cases and 23% of TB deaths (Table 1).

**Table 1:** Tuberculosis (TB) burden in older adults (≥ 65 years), global 2000 vs. 2023

Measure	2000	2023
Population ≥ 65 years (millions)	419.8	771.2
TB incidence cases ≥ 65 (thousands)	610	947
TB deaths ≥ 65 (thousands)	162	227
TB incidence rate (per 100,000)	145.3	122.7
TB mortality rate (per 100,000)	38.6	29.4

**Fig. 1:**
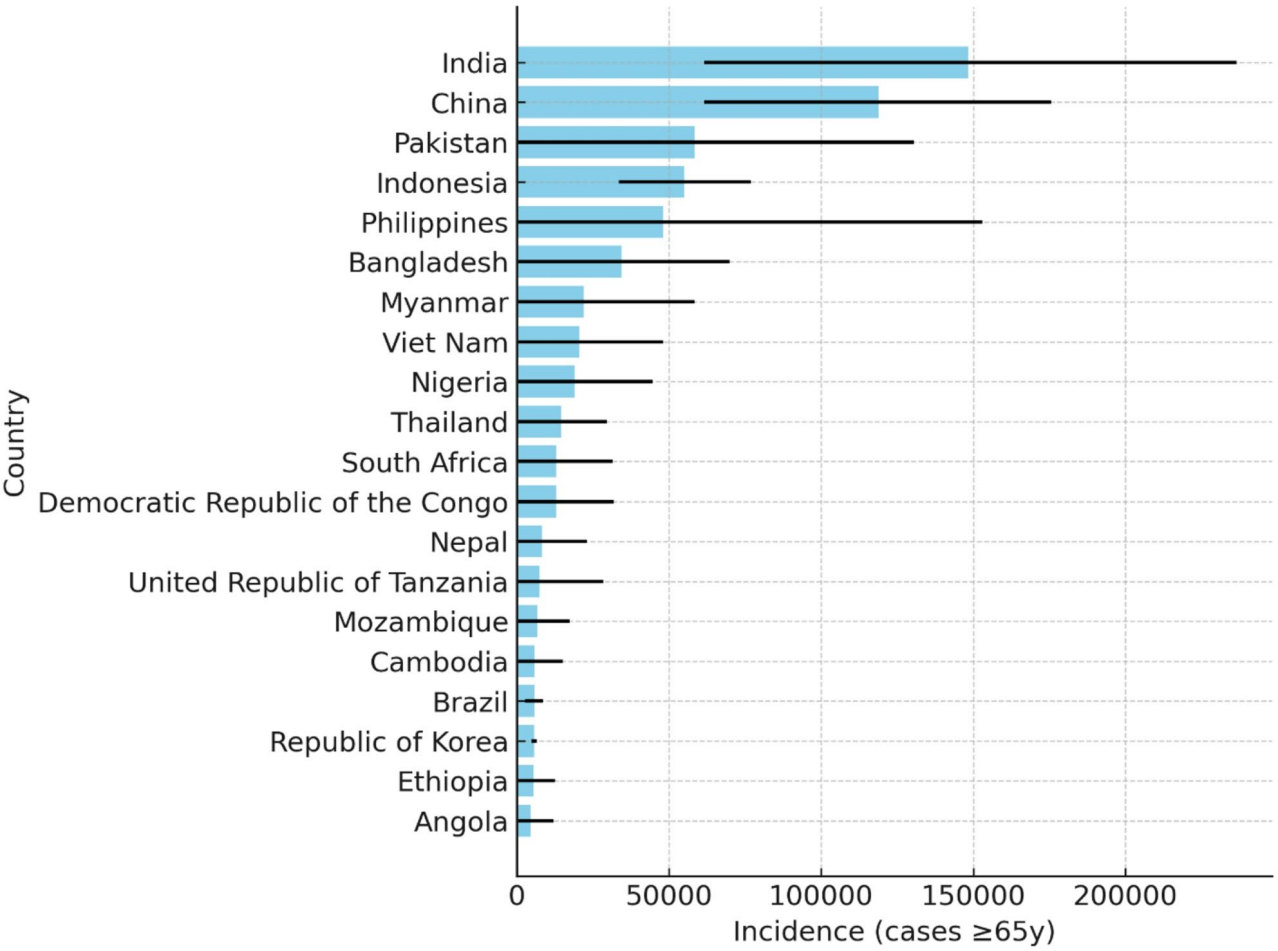
Top 20 countries with the highest estimated tuberculosis incidence among individuals aged ≥ 65 years in 2023. Bars represent mean estimated incidence per 100,000 population aged ≥ 65 years, and black lines indicate the 95% confidence intervals (CIs). Countries are ranked in descending order based on estimated incidence rates. Data were derived from the WHO Global Tuberculosis Database

Figure 1 illustrates the 20 countries with the highest estimated TB incidence among individuals aged ≥ 65 years in 2023. Incidence levels varied widely across nations, with especially high rates observed in parts of East Asia, the Pacific, and several European countries, underscoring substantial geographic heterogeneity in the burden of TB among older adults.

Long-term trend analysis (2000–2023) showed that TB incidence among adults ≥ 65 years decreased only modestly (from 145.3 to 122.7 per 100,000), whereas younger adults experienced substantially sharper declines, resulting in a widening age-related disparity over time. This divergence was most pronounced between 2010 and 2020 and briefly interrupted during the COVID-19 pandemic years (2020–2021), when temporary drops in case detection were observed in many countries.

When comparing TB rates between older adults and younger populations, the rate ratio of both TB incidence and TB-related mortality was significantly higher in individuals aged ≥ 65 years. Rate ratios for incidence and mortality were consistently elevated across all WHO regions, with the highest relative burden observed in the Western Pacific and South-East Asia regions (Table 2). Globally, the incidence RR for individuals aged ≥ 65 vs. individuals aged 15–64 years was 2.2 (95% CI: 2.0–2.4), and the mortality RR was 3.4 (95% CI: 3.1–3.8).

**Table 2:** Incidence and mortality rate ratios (RR) of tuberculosis in older adults (≥ 65 years) vs. individuals aged (15–64 years), 2023

WHO Region	Incidence RR (95% CI)	*p*-value	Mortality RR (95% CI)	*p*-value
Africa	1.8 (1.5–2.1)	< 0.001	2.4 (2.0–2.8)	< 0.001
Americas	2.1 (1.7–2.5)	< 0.001	3.0 (2.5–3.6)	< 0.001
Eastern Mediterranean	2.5 (2.1–3.0)	< 0.001	3.8 (3.2–4.4)	< 0.001
Europe	2.9 (2.4–3.5)	< 0.001	4.5 (3.8–5.3)	< 0.001
South-East Asia	1.7 (1.4–2.0)	< 0.001	2.2 (1.9–2.6)	< 0.001
Western Pacific	2.4 (2.0–2.8)	< 0.001	3.3 (2.8–3.9)	< 0.001
Global	2.2 (2.0–2.4)	< 0.001	3.4 (3.1–3.8)	< 0.001

**Fig. 2:**
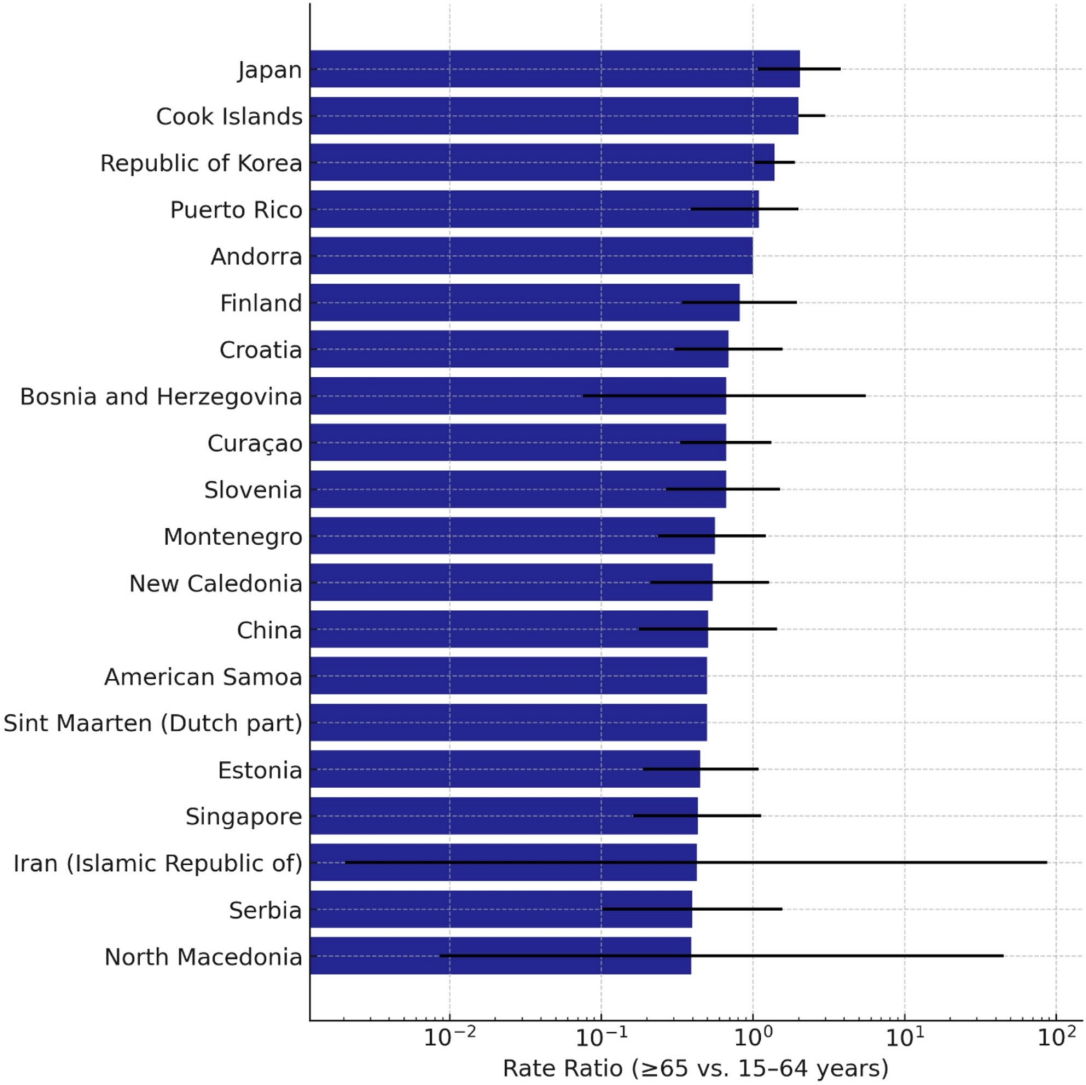
Population-based age-specific incidence rate ratios (RRs) of tuberculosis among individuals aged ≥ 65 years compared with individuals aged 15–64 years in 2023, stratified by country. Bars indicate the mean rate ratios, with error bars showing the 95% confidence intervals (CIs). Higher RR values indicate a disproportionately greater population-level TB burden among older adults relative to the WHO adult reference population.

At the country level, the magnitude of age-related differences varied widely. Japan, the Republic of Korea, and several European countries exhibited particularly high incidence rate ratios for older aged ≥ 65 versus individuals aged 15–64 years, while other high-burden countries showed smaller relative differences (Fig. 2). Several Eastern European countries demonstrated high RRs partly attributable to the regional MDR-TB epidemic.

**Fig. 3:**
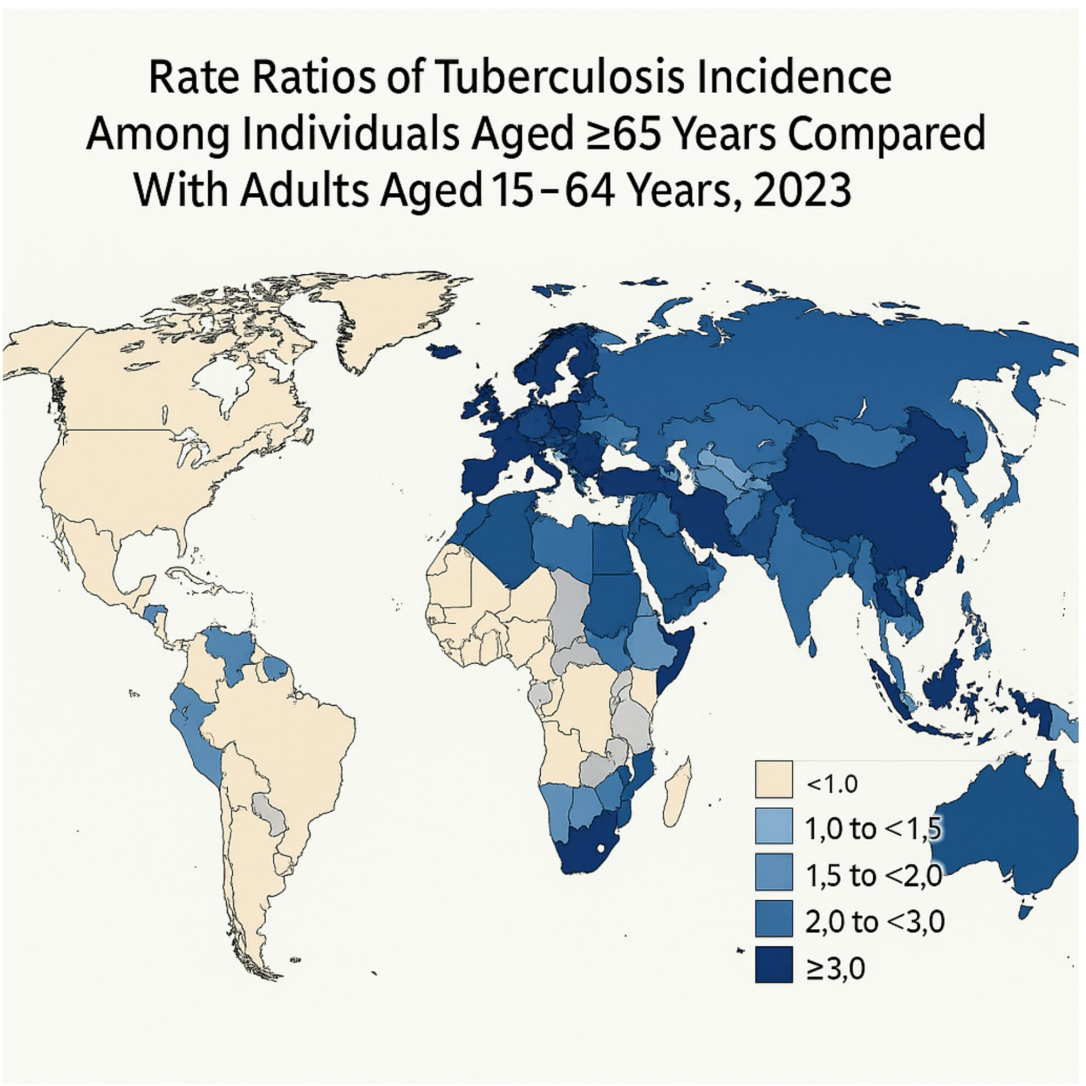
Global distribution of population-based age-specific incidence rate ratios (RRs) of tuberculosis among individuals aged ≥ 65 years compared with individuals aged 15–64 years in 2023. Countries are shaded according to the magnitude of the RR, with darker shading indicating a greater population-level TB burden among older adults. This map replaces the incidence-based version to better reflect age-specific disparities across countries

The global map (Fig. 3) displays rate ratios rather than raw incidence, revealing sharp contrasts between countries with rapidly ageing populations (e.g., Japan, Singapore, Italy) and those with younger demographic structures. Countries in South-East Asia, sub-Saharan Africa, and Eastern Europe demonstrated the highest incidence rates in 2023, often exceeding 150 cases per 100,000 population aged ≥ 65 years, with South-East Asia additionally influenced by the region’s high prevalence of diabetes and delayed TB detection, and sub-Saharan Africa affected by the HIV–TB co-epidemic.

**Fig. 4:**
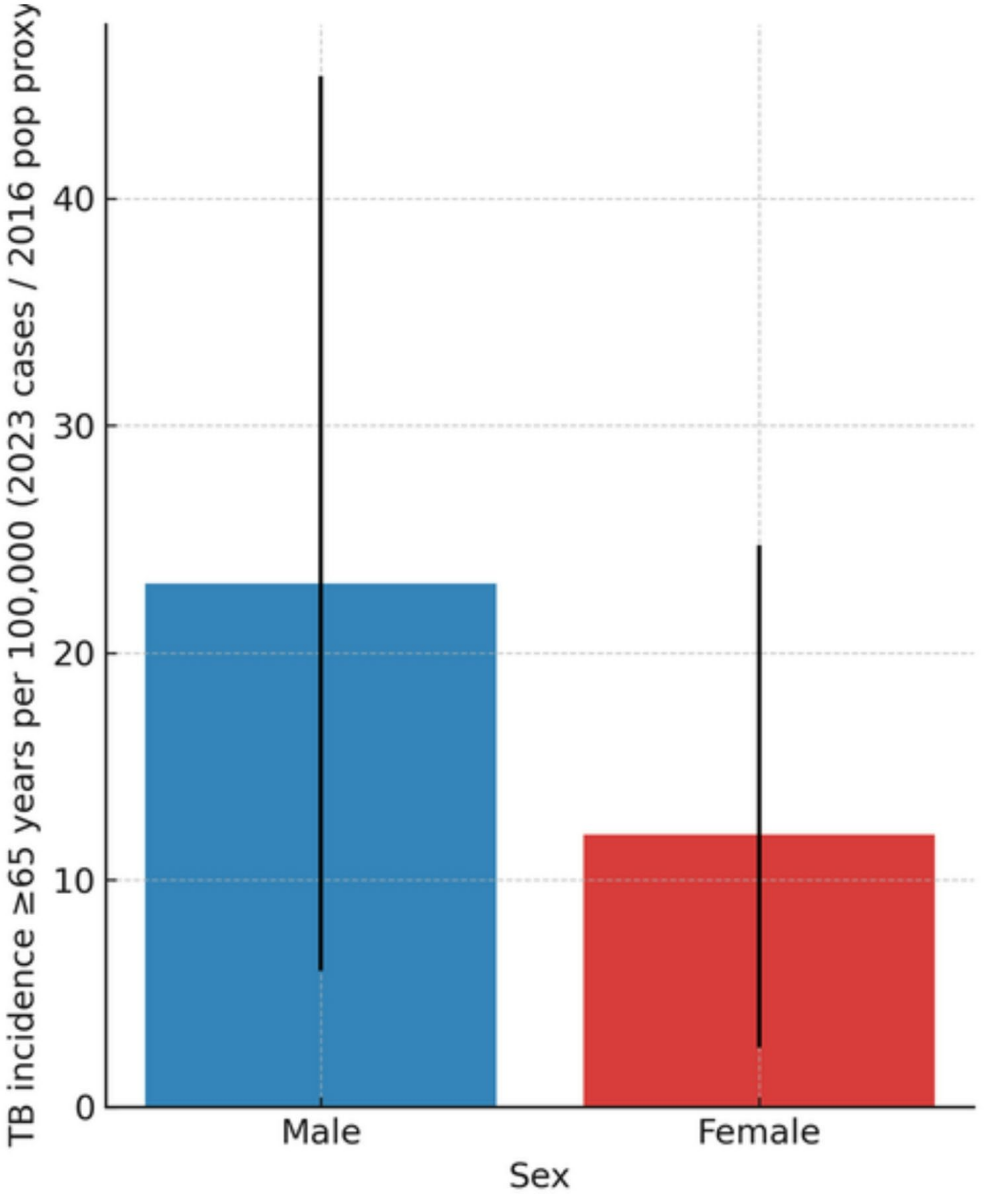
Sex-specific tuberculosis incidence among individuals aged ≥ 65 years in 2023. Blue and red bars represent the estimated incidence per 100,000 population for men and women, respectively. Error bars indicate the 95% confidence intervals (CIs), reflecting uncertainty in the estimated case counts

Sex-specific analyses revealed a clear excess of TB incidence among older men compared with older women. Globally, men aged ≥ 65 years had approximately double the incidence compared with women of the same age group, with overlapping but distinct 95% confidence intervals (Fig. 4). This sex disparity remained consistent across all WHO regions and persisted throughout the 2000–2023 period.

Overall, the results demonstrate that both demographic ageing and persistent epidemiological vulnerabilities contribute to a growing share of global TB burden among adults aged ≥ 65 years.

## Discussion

Tuberculosis (TB) remains a significant public health challenge among older adults (≥ 65 years), with incidence and mortality rates continuing to rise in many regions despite overall declines in younger populations [17–19]. Older adults are particularly susceptible due to immunosenescence, a higher prevalence of comorbidities such as diabetes, cardiovascular disease, and chronic pulmonary conditions, as well as delays in diagnosis that can result from atypical clinical presentations [20–22]. The proportion of TB cases occurring in this age group has been steadily increasing, largely driven by rapid global population ageing and improvements in TB control among younger adults, which shift the disease burden toward older populations [23–25]. Our trend analysis from 2000 to 2023 confirms that absolute TB cases and deaths in adults ≥ 65 years have risen substantially, even in regions where overall TB incidence has declined. The rate ratios reported in this study reflect population-level comparisons of tuberculosis incidence and mortality between age groups and should not be interpreted as individual-level risk estimates.

Sex-specific differences in TB burden are evident, with men consistently exhibiting higher incidence and mortality rates than women [21, 24]. This disparity may arise from differences in exposure, biological susceptibility, health-seeking behaviors, and comorbidity profiles, underscoring the importance of developing interventions that consider sex-specific risk factors and treatment needs [21, 26]. Socioeconomic factors, including poverty, limited access to healthcare, and living conditions that facilitate transmission, further exacerbate TB risk and contribute to delays in diagnosis and treatment [26–28]. Our findings show that these sex differences persist across all WHO regions and have remained stable over the 23-year period studied.

The emergence of multidrug-resistant TB (MDR-TB) and extensively drug-resistant TB (XDR-TB) represents a growing challenge, particularly among older adults who may have increased vulnerability to adverse drug effects, polypharmacy, and comorbidity-related complications [29–31]. Effective management of these resistant forms requires timely detection, appropriate treatment regimens, and close clinical monitoring to improve outcomes and prevent further transmission [29–31]. In Eastern Europe, high MDR-TB prevalence contributes significantly to the elevated burden observed among older adults, as reflected in the regional rate ratios.

Regional differences in TB burden among older adults are pronounced and reflect distinct epidemiologic and historical contexts. In sub-Saharan Africa, the HIV–TB co-epidemic continues to amplify risk among older adults, despite improving antiretroviral therapy coverage. In South-East Asia, high population density, delayed case detection, and an increasing prevalence of type 2 diabetes contribute to elevated TB incidence. In the Western Pacific region—particularly Japan and the Republic of Korea—the combination of rapidly ageing populations and historical TB transmission patterns has produced some of the highest age-specific incidence rate ratios globally. These findings align with the RR-based geographic patterns shown in Fig. 3.

Global TB control efforts face multiple challenges, including funding gaps, especially in low- and middle-income countries, limited healthcare infrastructure, and social determinants that increase vulnerability among older adults [32–34]. National policy implementation varies widely: while some countries have adopted age-targeted screening, long-term care facility surveillance, and intensified case finding, others lack geriatric-specific TB strategies. Addressing these challenges necessitates integrated public health approaches that combine medical, social, and policy interventions. Early detection through targeted screening, effective management of comorbidities, implementation of age- and sex-specific strategies, and provision of social and healthcare support are essential components for reducing TB incidence and mortality in older populations [17, 29, 35–37].

In summary, combating TB among older adults requires a comprehensive and holistic approach. Interventions should be evidence-based, context-specific, and tailored to the unique vulnerabilities of this population. Given the continued demographic expansion of the ≥ 65-year age group globally, TB among older adults will represent a growing share of the global TB burden in the coming decades. By integrating medical management, public health strategies, and social support, it is possible to mitigate TB burden, improve treatment outcomes, and reduce health disparities among older adults globally [17–37].

### Limitations

This study has several limitations. First, the analysis relies on WHO-reported tuberculosis case notifications and United Nations population estimates; underreporting, diagnostic gaps, and reporting delays—particularly in low-resource settings—may bias incidence and mortality estimates. Population estimates for older adults were based on the latest available UN age-specific data; more recent revisions are unlikely to substantially alter relative age-group comparisons. Second, the COVID-19 pandemic (2020–2022) substantially disrupted TB surveillance, healthcare access, and case detection worldwide, which may have led to temporary underestimation of TB incidence and mortality, including among older adults. Third, some countries lacked complete sex-specific population data for individuals aged ≥ 65 years, necessitating the use of proxy values from total population counts, which may have resulted in minor imprecision in sex-specific incidence estimates. Fourth, the study design is descriptive and ecological in nature; therefore, causal inferences regarding individual-level risk factors, comorbidities, or healthcare access cannot be made. Fifth, substantial demographic changes between 2000 and 2023—particularly the rapid global expansion of the population aged ≥ 65 years —may influence absolute case counts and complicate comparisons across time and regions. Sixth, differences in national age structures may affect rate ratios, as countries with rapidly ageing populations (e.g., Japan or Italy) may exhibit higher age-specific rate ratios independent of underlying transmission dynamics. Data on multidrug-resistant TB (MDR-TB) and extensively drug-resistant tuberculosis (XDR-TB) among older adults were limited, precluding a detailed assessment of drug resistance patterns in this age group. Finally, regional heterogeneity in diagnostic practices, reporting completeness, and population-based screening strategies may contribute to the observed geographic variation. Despite these limitations, this study provides one of the most comprehensive global assessments of tuberculosis burden among adults aged ≥ 65 years to date.

## Conclusions

Tuberculosis remains a significant public health challenge among older adults aged ≥ 65 years. Over the period 2000–2023, the global burden in this age group has increased in absolute numbers, partly as a result of population ageing, and the rate ratio compared with younger adults remains higher across all WHO regions. Older men are disproportionately affected compared with women. Tailored public health strategies, including targeted screening, age- and sex-specific interventions, and integration of comorbidity management, are critical to reduce incidence and mortality. Given the pronounced regional heterogeneity—driven by factors such as the HIV–TB co-epidemic in sub-Saharan Africa, high diabetes prevalence in South-East Asia, and MDR-TB in parts of Eastern Europe—country-specific approaches are essential. Strengthening TB surveillance in older populations and improving access to care can mitigate health disparities and improve treatment outcomes globally. As global populations continue to age, focused strategies addressing the needs of older adults will be increasingly important for achieving long-term TB control.

## Data Availability

All data used in this study are publicly available:• WHO Global TB Database: [https://www.who.int/teams/global-programme-on-tuberculosis-and-lung-health/data] • UN World Population Prospects 2016 revision: [https://population.un.org/wpp/]. All analyses were conducted using these publicly accessible sources without modification.
